# Clinical Manifestations, Laboratory Findings, and Treatment Outcomes of SARS Patients

**DOI:** 10.3201/eid1005.030640

**Published:** 2004-05

**Authors:** Jann-Tay Wang, Wang-Huei Sheng, Chi-Tai Fang, Yee-Chun Chen, Jiun-Ling Wang, Chong-Jen Yu, Shan-Chwen Chang, Pan-Chyr Yang

**Affiliations:** * National Taiwan University Hospital, Taipei, Taiwan

**Keywords:** severe acute respiratory syndrome, C-reactive protein, intravenous immunoglobulin

## Abstract

Clinical and laboratory data on severe acute respiratory syndrome (SARS), particularly on the temporal progression of abnormal laboratory findings, are limited. We conducted a prospective study on the clinical, radiologic, and hematologic findings of SARS patients with pneumonia, who were admitted to National Taiwan University Hospital from March 8 to June 15, 2003. Fever was the most frequent initial symptom, followed by cough, myalgia, dyspnea, and diarrhea. Twenty-four patients had various underlying diseases. Most patients had elevated C-reactive protein (CRP) levels and lymphopenia. Other common abnormal laboratory findings included leukopenia, thrombocytopenia, and elevated levels of aminotransferase, lactate dehydrogenase, and creatine kinase. These clinical and laboratory findings were exacerbated in most patients during the second week of disease. The overall case-fatality rate was 19.7%. By multivariate analysis, underlying disease and initial CRP level were predictive of death.

Severe acute respiratory syndrome (SARS) is a new infectious disease, first recognized in November 2002 (*1*). SARS has spread rapidly around the world: >8,400 cases have been reported from 30 countries on five continents (*2*,*3*).

Previous reports have described some major clinical findings of SARS, including the temporal progression of clinical symptoms and chest radiography, the outcomes, suggested treatment protocol, and risk factors for death (*4*,*5*). However, data are still limited on the temporal progression of abnormal laboratory findings, such as leukopenia, lymphopenia, thrombocytopenia, elevated lactate dehydrogenase (LDH), elevated aspartate aminotransferase (AST), elevated alanine aminotransferase (ALT), elevated creatine kinase (CK), and elevated C-reactive protein (CRP) and the roles each of these plays in predicting outcomes and complications. Although So et al. have suggested a treatment protocol with the emphasis on early use of high-dose steroids (*6*), whether this treatment is better than others is unclear.

We conducted a prospective study on the clinical, radiologic, and hematologic findings of SARS patients with pneumonia, who were admitted to National Taiwan University Hospital (NTUH) from March 8 to June 15, 2003. Most of these patients were treated following a standard treatment protocol, different from that suggested by the study group in Hong Kong (*6*). We report on the clinical features of our SARS patients with pneumonia, with emphasis on temporal progression of laboratory findings, treatment outcome, and risk factors for poor prognosis.

## Methods

### Setting

NTUH is a 2000-bed, university-affiliated medical center located in northern Taiwan. The center provides both tertiary and primary care for patients. It was also the primary hospital caring for SARS patients during the SARS outbreak in Taiwan.

### Patient Description and Treatment Protocol

All patients who fulfilled the revised World Health Organization (WHO) definition of probable SARS (*7*) in whom pneumonia developed and who received treatment at NTUH during March 8 to June 15, 2003, were enrolled in this study. Except for the first patient, who did not receive any of the following treatment, and the second, third, and fourth patients, who received steroid in the first week of their disease, all patients received treatments that conformed to the guideline described here. Oral ribavirin was prescribed soon after the diagnosis of SARS was made; the loading dose was 2,000 mg followed by 1,200 mg per day if the body weight was >75 kg, or 1,000 mg per day if the body weight was <75 kg. This treatment lasted 10 days unless adverse effects developed. Antimicrobial agents for community-acquired pneumonia, either moxifloxacin alone or ceftriaxone plus azithromycin, were administered at the same time. Methylprednisolone was usually administered in the second week of the disease if any of the following occurred: a flare of fever, progression of clinical symptoms (such as dyspnea or diarrhea), a surge or resurge of CRP level, or rapid deterioration of chest radiographic findings (development of new infiltration). Methylprednisolone was indicated in the first week of disease only if clinical symptoms or laboratory abnormalities (such as elevated CK, LDH, CRP) worsened rapidly, and rapidly progressed abnormalities were found on chest radiograph. The dosage was 2 mg/kg/day for 5 days, and then it was tapered off. Pulse therapy with methylprednisolone, 500 mg/day for 3 days, was used if there was major disease progress under the standard regimen. Intravenous immunoglobulin (IVIG) was administered if severe leukopenia (<2 x 10^9^/L), thrombocytopenia (<100 x 10^9^/L), or both occurred, or if lesions on chest radiography progressed rapidly in the first week of disease. The dosage of IVIG was 1 g/kg/day for 2 days. Once patients were intubated and supported by a mechanical ventilator, respiratory care followed the principles suggested for managing acute respiratory distress syndrome (*8*).

### Laboratory Examination

The etiologic workup included the sputum Gram stain and acid-fast stain, sputum culture for bacteria, sputum chlamydial antigen, throat swab for virus isolation, urine pneumococcal antigen, and urine legionella antigen. We tested antibody reactions of both acute- and convalescent-phase serum specimens, 4 weeks apart, for *Mycoplasma, Chlamydia,* influenza virus, parainfluenza virus, adenovirus, coxsackievirus, respiratory syncytial virus, and SARS-related coronavirus (SARS-CoV). We also took throat swabs for reverse transcription–polymerase chain reaction (RT-PCR) for SARS-CoV. The other routine laboratory tests, such as the hemogram, serum AST, ALT, CK, LDH, and CRP level, were examined every other day during hospitalization. A chest radiography was also performed every other day during hospitalization.

### Infection Control Measures

Once a patient was diagnosed as having SARS, he or she was sent to a negative-pressure ventilated room immediately. No visitor or family member was allowed to enter this room. All healthcare workers caring for SARS patients were asked to adhere strictly to contact and air-borne precautions. Before entering isolation rooms to care for SARS patients, all healthcare workers washed their hands and put on personal protective equipment, including gowns, gloves, N95 respirators, goggles, and face shields. After caring for SARS patients, such workers were to take off the personal protective equipment in the anteroom and wash their hands before leaving the isolation room. The health of healthcare workers who had any contact with SARS patients or their environments was monitored daily for 14 days after the last exposure. Once fever developed in a worker, he or she was immediately hospitalized and placed in isolation in a specially designated ward.

### Data Collection

A standard case report form modified from one designed by the Centers for Disease Control and Prevention for SARS was used to collect demographic and clinical data (*9*). Severity of underlying disease was classified by using the modified risk stratification proposed by McCabe: rapidly fatal (death expected within 1 year), ultimately fatal (death expected within 5 years), or nonfatal (death expected >5 years or no underlying disease) (*10*).

### Statistics

All statistical analysis was performed with SPSS version 10.0 (SPSS, Chicago, IL). Logistic regression was used for univariate and multivariate analysis. Continuous variables were compared with the *t* test. Categorical variables were compared by using the Fisher exact test. A p value < 0.05 was considered significant.

## Results

During the study period, 76 patients were enrolled. Their demographic and clinical data are detailed in Table 1. The male-to-female ratio was 34:42. Their age was 24–87 years (median 46.5 years). Twenty-four patients had various underlying diseases, including cardiovascular disorders in 13 patients, diabetes mellitus in 10, hepatobiliary disorders in 6, history of cerebrovascular accidents in 3, chronic renal diseases in 2, pulmonary fibrosis in 1, history of intravenous drug abuse in 1, and adrenal insufficiency in 1. Fourteen of these 24 patients had underlying diseases classified as rapidly fatal (diabetes mellitus, ischemic heart disease, plus congestive heart failure in four patients; diabetes mellitus, ischemic heart disease, plus cerebrovascular accident with being bedridden in three; diabetes mellitus, ischemic heart disease, plus end-stage renal disease in two; diabetes mellitus plus decompensated liver cirrhosis in one; and ischemic heart disease plus massive ischemic bowel in one) or ultimately fatal (severe pulmonary fibrosis in one, ischemic heart disease in two). Most frequent initial symptoms were fever, cough, myalgia, dyspnea, diarrhea, and rigor. Three of the 24 patients who had diarrhea had previously received various antimicrobial agents. The duration from symptom onset to a patient’s visiting NTUH was 1–12 days (median 3 days). The initial laboratory data are detailed in Table 2. Abnormalities on chest radiography suggesting pneumonia were found in 56 of the 76 patients. Lesions were found in one lobe in 33 patients, two lobes in 15 patients, three lobes in 4 patients, four lobes in 2 patients, and five lobes in 2 patients. Abnormalities visible on chest radiography developed in the other 20 patients after admission. The duration from disease onset to the time when abnormalities on the chest radiography were first noted was 1–12 days (median 4 days).

**Table 1:** Demographic data and initial clinical signs and symptoms of 76 patients with probable SARS^a^

Data	No. of cases (%)
Sex	
Male	34 (44.7)
Female	42 (55.3)
Age (y) (range, median)	24–87 (46.5)
Underlying disease (no.)	
Nonfatal	62 (81.6)
No underlying disease	52 (68.4)
Mild underlying disease	10 (13.2)
Ultimately fatal	3 (3.9)
Rapidly fatal	11 (14.5)
Initial symptoms	
Fever	76 (100)
Cough	47 (61.8)
Myalgia	37 (48.7)
Dyspnea	31 (40.8)
Diarrhea	24 (31.6)
Rigor	23 (30.3)
Headache	14 (18.4)
Nausea	9 (11.8)
Sore throat	7 (9.2)
Vomiting	3 (3.9)
Rhinorrhea	2 (2.6)

^a^SARS, severe acute respiratory syndrome.

**Table 2:** Initial laboratory data of 76 patients with SARS^a^

Laboratory parameters (normal range)	Mean ± standard deviation	No. (%) of patients with abnormal data
Leukocyte counts (4–9 x 10^9^/L)	6.0 ± 2.9	
Leukopenia		15 (19.7)
Lymphopenia^b^		49 (64.5)
Platelet counts (150–450 X 10^9^/L)	159.7±54.0	
Thrombocytopenia		35 (46.1)
AST (<35 U/L)	36.7±20.0	24^c^ (35.3)
ALT (<35 U/L)	27.5±20.4	11^d^ (23.9)
LDH (<460 U/L)	597.8±426.0	9^e^ (56.3)
CK (<190 U/L)	216.5±444.3	17^f^ (26.1)
CRP (<0.8 mg/dL)	3.9±3.6	53^g^ (77.9)

^a^SARS, severe acute respiratory syndrome; AST, aspartate aminotransferase; ALT, alanine aminotransferase; LDH, lactate dehydrogenase; CK, creatine kinase; CRP, C-reactive protein. ^b^Defined as <1 X 10^9^/L. ^c^Only 68 patients had been tested. ^d^Only 46 patients had been tested. ^e^Only 16 patients had been tested. ^f^Only 65 patients had been tested. ^g^Only 68 patients had been tested.

During hospitalization, 69 patients (90.8%) had respiratory distress and needed oxygen supplements. The duration from disease onset to severe respiratory distress was a mean of 9.8 ± 3.0 days (standard deviation [SD]). Endotracheal intubation with ventilator support was indicated for 26 patients, but 3 patients refused intubation. Among the 23 intubated patients, the duration from disease onset to intubation was 8.4 ± 3.3 (SD) days. Eight of these 23 patients were successfully extubated 12.1 ± 6.1 (SD) days later. Twelve of the 23 patients died, and 3 patients remained intubated at the end of the study because of marked lung fibrosis.

Thirty-one patients (40.8%) experienced exacerbation of diarrhea after admission. All had received various antimicrobial agents since hospitalization. The duration from disease onset to severe diarrhea was 8.9 ± 4.7 (SD) days.

During the disease course, leukopenia, lymphopenia, and thrombocytopenia were found in 40, 72, and 61 patients, respectively. Elevation of AST and ALT was noted in 66 and 59 patients, respectively. Elevation of serum LDH, CK, and CRP levels was found in 73, 34, and 71 patients, respectively. The laboratory values of the various parameters listed above typically peaked in severity in the second week of illness (Table 3). The Figure demonstrates the relationships between the time points when several clinical and laboratory parameters became most severe.

**Table 3:** Data on most severe abnormal laboratory parameters of 76 SARS patients during their disease course^a^

Laboratory parameters (no. of patients, %)	Mean ± SD of most severe data (unit)	Duration from disease onset to most severe data noted
Leukopenia (40, 52.6)	2.5 ± 0.7 (x 10^9^/L)	7.5 ± 2.4 days
Lymphopenia (72, 94.7)	0.6 ± 0.3 (X 10^9^/L)	7.0 ± 2.5 days
Thrombocytopenia (61, 80.3)	102.3 ± 31.3 (X 10^9^/L)	6.9 ± 2.0 days
Elevated AST (66, 86.9)	142.0 ± 323.6 (U/L)	10.3 ± 4.6 days
Elevated ALT (59, 77.6)	103.7 ± 132.2 (U/L)	13.3 ± 5.0 days
Elevated LDH (73, 96.1)	1323.8 ± 1487.2 (U/L)	10.8 ± 3.9 days
Elevated CK (34, 44.7)	12165.7 ± 58226.9 (U/L)	7.8 ± 4.2 days
Elevated CRP (71, 93.4)	7.1 ± 4.0 (mg/dL)	8.5 ± 3.0 days

^a^SARS, severe acute respiratory syndrome; SD, standard deviation.

**Figure:**
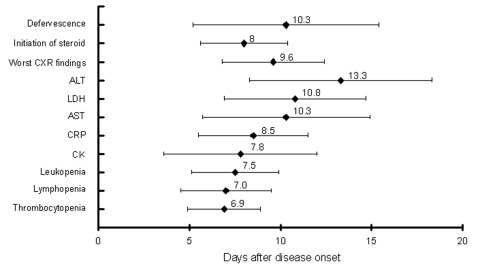
The time relationships between the time points of defervescence, initiation of steroid, and when chest radiographic finding as well as various laboratory parameters became most severe. Mean and standard deviation (days) are presented. CXR, chest radiography; ALT, alanine aminotransferase; LDH, lactate dehydrogenase; AST, aspartate aminotransferase; CRP, C-reactive protein; CK, creatine kinase.

New lesions visible on chest radiography developed in 64 patients during hospitalization, including new lesions limited to one lung lobe in 21 patients, two lobes in 13, three lobes in 16, four lobes in 7, and five lobes in 7. The duration from disease onset to the most severe chest radiography findings was 9.6 ± 2.9 (SD) days.

Sputum Gram stain and acid-fast stain, sputum culture for bacteria, sputum chlamydial antigen, throat swab for virus isolation, and urine legionella and pneumococcal antigen tests were available for all 76 patients; all results were negative. Paired serum specimens were available for 41 patients. Tests for antibody reaction to *Mycoplasma, Chlamydia,* influenza virus, parainfluenza virus, adenovirus, coxsackievirus, and respiratory syncytial virus were negative. Of these 41 patients, immunoglobulin (Ig) G antibody to SARS-CoV was detected by immunofluorescent assay in 38 patients (92.7%). Twenty-six (34.2%) of the total 76 patients had positive results on throat swab RT-PCR for SARS-CoV. Among the 50 patients whose throat swab results were negative for SARS-CoV by RT-PCR, 28 seroconverted to SARS-CoV. The other 22 patients, who had no direct microbiology or serologic evidence for SARS-CoV infection at the time the study ended, had clinical courses compatible with those of probable SARS and clear relationships as well as exposures to the initial immigrant clusters and the later intrahospital outbreaks in Taiwan (*11*). Their diagnoses of SARS had also been confirmed by a committee of the Center for Disease Control (Taiwan).

The first patient did not receive any treatment specific for SARS. Five patients did not receive ribavirin treatment because its use was contraindicated for them because of conditions such as cardiac arrhythmia and cardiomyopathy. Seven patients did not receive steroids because their cases were rapidly fatal after diagnosis. These seven patients died mainly because of their underlying diseases, especially cardiac events. Their SARS cases did not progress to the point that steroids were indicated according to our treatment protocol.

Eight patients received pulse steroid therapy for progressive clinical conditions, with the usual dosage of steroids. Forty patients received IVIG infusion for severe cytopenia (thrombocytopenia, leukopenia, or both) (22 patients in total) or marked local progression of pulmonary lesions on chest radiography in the first week of disease (18 patients). Those 22 patients who received IVIG for severe cytopenia did not receive steroids while on IVIG; their leukocyte counts were 2.6 ± 1.2 x 10^9^/L and 4.3 ± 2.8 x 10^9^/L, before and after the use of IVIG, respectively (p = 0.014, by paired *t* test). Their platelet counts were 104 ± 35 x 10^9^/L and 141 ± 46 x 10^9^/L, before and after IVIG, respectively (p = 0.002, by paired *t* test).

Various complications developed in 18 patients during hospitalizations, including rhabdomyolysis, peripheral neuropathy, acute renal failure, and fungal or bacterial superinfection (Table 4). Among the 18 nosocomial infections, 6 were bloodstream infections; 3 of these were caused by enterococci, 2 by methicillin-resistant *Staphylococcus epidermidis*, and 1 by methicillin-resistant *S. aureus*. Eleven episodes were lower respiratory infections, diagnosed by the existence of new infiltration on chest radiography, purulent sputum, phagocytosis of bacteria by neutrophils in sputum (indicated by sputum Gram stain), positive sputum culture for bacteria, and response to effective antimicrobial agents. The last episode was a catheter-related infection caused by *Candida parapsilosis*.

**Table 4:** Complications found in 76 SARS patients^a^

Complication	No. of patients (%)
Rhabdomyolysis	4 (5.3)
Peripheral neuropathy	5 (6.6)
Acute renal failure	3 (3.9)
Gastrointestinal bleeding	2 (2.6)
Acute myocardial infarction	1 (1.3)
Bacteria superinfection	10 (13.2)
*Candida parapsilosis* superinfection	1 (1.3)
MRSA	4 (5.3)
MRSE	2 (2.6)
Enterococci	3 (3.9)
*Acinetobacter baumannii*	3 (3.9)
*Klebsiella pneumoniae*	2 (2.6)
*Enterobacter cloacae*	1 (1.3)
*Serratia marcescense*	1 (1.3)
Total	18 (23.7)

^a^SARS, severe acute respiratory syndrome; MRSA, methicillin-resistant *Staphylococcus aureus*; MRSE, methicillin-resistant *Staphylococcus epidermidis*. Some patients experience more than one complication.

The overall death rate was 19.7% (15/76). Among the 14 patients whose underlying diseases were classified as ultimately fatal or rapidly fatal, the rate of death was 78.6% (11/14). For the other 62 patients with mild underlying diseases or without underlying disease, the death rate was 6.5% (4/62). The time from disease onset to death in these 15 patients was 4–42 days (median 12 days). For the 26 patients whose clinical conditions indicated endotracheal intubation with ventilator support, the rate of death was 57.7% (15/26). When we used the logistic regression model for univariate analysis, age, underlying disease (nonfatal versus ultimately or rapidly fatal), initial CRP level, initial absolute neutrophil count (ANC), peak CK level, and peak CRP level were predictors of death. Age, sex, underlying disease, initial chest radiographic findings, initial CRP level, initial ANC, peak CK level, lowest lymphocyte count, worst chest radiographic findings, peak LDH level, and peak CRP level were the predictive factors for respiratory failure (Table 5). However, when we used logistic regression for multivariate analysis, underlying disease and initial CRP level were the only two factors significantly predictive for death (odds ratio [OR], 83.333 and 1.447 every 1 mg/dL increase, respectively; p < 0.001 and p = 0.006, respectively), and age, initial CRP level, and worst chest radiographic findings were predictive for respiratory failure (OR 1.076, 1.419 every 1 mg/dL, and 2.501 every one-lobe involvement, respectively; p = 0.01, p = 0.01, and p = 0.006, respectively). For the 65 patients who received steroids as the treatment protocol described above, 3 remained febrile and needed further pulse steroid therapy after the use of methylprednisolone with the dosage of 2 mg/kg/day. For the other 62 patients who became afebrile after steroid was administered, 12 had rebound of fever 2–3 days after the temporary defervescence. Seven of the 12 patients became afebrile again and had no fever 1–2 days after the transient rebound of fever without specific intervention. The other five patients received further pulse steroid therapy to control the fever and exacerbated clinical symptoms. For those 62 patients alive at the end of this study, the time to defervescence after disease onset was 10.3 ± 5.1 days. Major lung fibrosis directly caused by SARS developed in 6 of these 62 patients; this condition resulted in exertional dyspnea in 2 patients, oxygen-supplement dependence in 1 patient, and respiratory failure in 3 patients. By the end of the study, 58 of the 62 patients had been successfully discharged from NTUH. The other four patients, including three patients who had respiratory failure and one who was dependent on oxygen supplement, remained hospitalized. The duration of follow-up for these four patients was >4 weeks.

**Table 5:** Univariate analysis of factors predictive for death and respiratory failure (logistic regression)^a^

Factors	Odds ratio		p value		
	Mortality	ResF	Mortality	ResF	
Age (every 1 y)^b^	1.093	1.082	<0.001	<0.001	
Sex (male to female)	3.086	2.841	0.064	0.036	
Severe underlying disease^c^	3.973	3.023	<0.001	<0.001	
Initial CRP level^b,c^ (every 1 mg/dL)	1.300	1.391	0.004	<0.001	
Initial ANC count (every 0.1x10^9^/L)	1.022	1.031	0.020	0.006	
Initial lymphocyte count (every 0.1x10^9^/L)	0.091	0.916	0.188	0.162	
Initial platelet count (every 100x10^9^/L)	0.668	0.565	0.485	0.248	
Initial CXR findings (every one-lobe involvement)	1.482	1.941	0.154	0.025	
Peak CRP level (every 1 mg/dL)	1.193	1.385	0.033	<0.001	
Lowest lymphocyte count (every 0.1x10^9^/L)	0.801	0.804	0.079	0.032	
Peak LDH level (every 100 U/L)	1.132	1.160	0.124	0.008	
Peak CK level (every 1 U/L)	1.093	1.082	<0.001	<0.001	
Lowest platelet count (every 100x10^9^/L)	0.724	0.541	0.608	0.261	
Worst CXR findings (every one-lobe involvement) ^a^	1.505	2.147	0.064	<0.001	

^a^ResF, respiratory failure; CRP, C-reactive protein; ANC, absolute neutrophil count; CXR, chest radiography; LDH, lactate dehydrogenase; CK, creatine kinase. ^b^Factors that remained statistically significant after multivariate analysis for respiratory failure. ^c^Factors that remained statistically significant after multivariate analysis for death.

## Discussion

Our study of 76 patients with probable SARS with pneumonia demonstrated a high case-fatality rate (19.7%), especially in patients with major underlying diseases and high initial CRP levels. Those patients who needed endotracheal intubation with ventilator support during their hospitalization also had a high rate of death (57.7%). Various complications developed in a high proportion of patients (23.7%) during their disease.

The yield rate of RT-PCR assay for SARS-CoV was lower (34.2%) than in a previous report (*5*). This finding might be because only throat swabs, not nasopharyngeal aspirations or stools, were obtained for RT-PCR in the present study.

As in previous reports from other areas (*4*,*5*), fever was the most frequent initial symptom in our cases. Compared to those previous reports, more patients in our case series initially had diarrhea (31.6% vs. 1%–19.6%). Therefore, according to our observations, diarrhea may be also considered as an early symptom and clue for SARS. In addition, 18 patients had initial symptoms of diarrhea when fever occurred. Gastrointestinal tract should be considered as another important primary infection site of SARS-CoV.

A previous study reported the temporal progression of clinical and radiologic findings in SARS patients and indicated that several parameters would become more severe in the second and third week of disease (*5*). Our study had similar findings. Although the exacerbation of diarrhea might be due to the use of antimicrobial agents, the diarrhea improved subsequently without their change or discontinuation. Therefore, exacerbation of diarrhea is more likely due to SARS itself. Our study also demonstrates that most patients’ abnormal laboratory findings may become more severe in the second week of disease (Table 3 and Figure).

Our treatment protocol was somewhat different from that suggested by So et al. (*6*). The timing of steroids was modified according to our experiences in treating the second, third, and fourth patients, whose exacerbation of oxygen demand and chest radiography lesions were not prevented by early steroid use. Moreover, steroids are immunosuppressive. A previous study pointed out that the viral load of SARS Co-V in SARS patients arrived at peak levels at approximately day 10 of disease (*5*). Steroids were used as an adjunctive therapy for infectious diseases to reduce the severity of inflammatory damage that could occur in the later stage of disease (*12*). Using steroids was also a risk factor for subsequent nosocomial infection (*13*). For all these reasons, we delayed the use of steroids. Among the 65 patients who received steroids as the treatment protocol, 15 (23.1%) had rebound or persistence of fever after initial steroid use. This fever rebound is less frequent than in prior reports (43.3%–85.3%) (*5*,*6*). However, the overall death rate in our study was similar to that reported from Hong Kong (7%–20.9%) (*3*,*5*). Comparing the treatment results of our study and previous ones is difficult because of different case definitions, patient backgrounds, and disease severity, as well as obscure descriptions about complications in SARS patients in previous reports. All our patients had severe cases. Therefore, the best timing of starting steroid usage and the total duration of steroid usage in SARS patients to improve treatment outcome remain unclear and need further study.

Hemophagocytotic syndrome was documented in our second patient by bone marrow biopsy (*14*). Her initial clinical signs and symptoms included fever, severe leukopenia, and thrombocytopenia. Her hemophagocytotic syndrome was relieved after using IVIG. This treatment was suggested because of its immune modulating effect (*15*). The other 21 patients in whom severe leukopenia or thrombocytopenia developed in the first week of disease also received IVIG therapy empirically. IVIG appeared effective for controlling leukopenia and thrombocytopenia: after infusion, the patients’ leukocyte and platelet counts increased to a significantly higher level (p = 0.002). The increase of leukocyte and platelet counts might have prevented some further complications directly resulting from severe leukopenia and thrombocytopenia, such as infection and tendency to bleed. Although we had no control group, we believe that IVIG may play a role in treating selected SARS patients.

Advanced age, co-existing conditions, high peak LDH level, and high initial ANC count had been reported as factors that predict poor prognosis for SARS patients (*4*,*5*,*16*). By univariate analysis, many parameters predicted death or respiratory failure (Table 3). However, by using the logistic regression model for multivariate analysis, severe underlying disease and high initial CRP level were the only two factors that predicted death; age, initial CRP level, and worst chest radiographic findings predicted respiratory failure. The role of CRP in predicting the outcome of SARS patients has not been discussed in previous studies (*4*,*5*,*16*). The discovery of CRP was reported in 1930 by Tillet and Francis (*17*). CRP parallels the severity of inflammation or tissue injury and is a useful marker for disease, response to therapy, and ultimate recovery (*18*,*19*). Although initial CRP level was not available in eight patients in this cohort, our findings suggest that CRP also parallels well with the severity and outcome of SARS patients. Age and underlying disease were strongly correlated: all our patients with severe underlying disease were older (age >65 years). In a statistical model, these two factors might interfere with each other and lead to the conclusion that age, not underlying disease, was an independent risk for respiratory failure; however, the opposite was true. The worst chest radiographic finding outlined the most severe extent of impaired lung function. This finding might explain why it was an independent factor for respiratory failure. However, other conditions unrelated to pulmonary condition, such as underlying disease or complications during hospitalization, contributed to death. These findings might explain why worst chest radiographic finding was not an independent factor for death.

Forty-two patients in this cohort were admitted to NTUH through the emergency department, which has no facility to check serum LDH level. Also, during intrahospital SARS outbreaks (*11*), heavy clinical loads and frequent bed transfers made it difficult for the primary care physician to collect laboratory data as the schedule described above. Therefore, initial serum LDH level and CRP level were available for only 16 and 68 patients, respectively; thus, initial LDH level could not be factored into our analyses. Since both CRP and LDH are markers of inflammation, whether the initial LDH level is also an independent risk factor for death or respiratory failure needs further study.

Complications during the disease courses of SARS patients have been seldom or obscurely discussed previously (*4*,*5*,*16*). Acute renal failure, which might be more likely caused by methicillin-resistant S*. aureus* infection and rhabdomyolysis, was found in three patients. Acute myocardial infarction occurred in a patient who had been diagnosed with long-standing coronary artery disease. Gastronintestinal bleeding, which might be due to critical illness, occurred in two patients. Rhabdomyolysis has been reported to be associated with viral infection (*20*,*21*). Our observation suggests that SARS Co-V infection might also be associated with this complication (*22*). Although peripheral neuropathy had also been reported with viral infection (*23*), neuropathy caused by steroids or acute illness should also be considered contributing causes in our four patients with neuropathy (*24*,*25*).

Eleven patients had bacterial or fungal superinfection during hospitalization. All nosocomial infections occurred while patients were intubated and supported with a mechanical ventilator (p < 0.001 by Fisher exact test). The nosocomial infection rate among SARS patients was 237 per 1,000 discharges, which was much higher than that of all patients at NTUH (49 per 1,000 discharges) (*26*). Steroid use and more severe clinical conditions than usual patients, such as higher rate of respiratory failure, might be the reasons.

Our study shows that SARS has an overall complication rate of 23.7% and case-fatality rate of 19.7%. Clinical symptoms and abnormal radiographic and laboratory findings might become most severe in the second week of disease. In addition to ribavirin and steroids, IVIG may play a role in treating selected patients. Underlying disease and initial CRP level were the two independent predictors of death; age, initial CRP level, and worst chest radiographic finding were the three independent factors predicting respiratory failure for adult SARS patients.
